# Ecotoxicity and fate of a silver nanomaterial in an outdoor lysimeter study

**DOI:** 10.1007/s10646-017-1805-4

**Published:** 2017-05-25

**Authors:** Karsten Schlich, Martin Hoppe, Marco Kraas, Elke Fries, Kerstin Hund-Rinke

**Affiliations:** 1 Fraunhofer Institute for Molecular Biology and Applied Ecology, Auf dem Aberg 1, Schmallenberg, 57392 Germany; 2 Federal Institute for Geosciences and Natural Resources, Stilleweg 2, Hannover, 30655 Germany

**Keywords:** Silver nanomaterials (AgNM), Fate, Ecotoxicity, Outdoor lysimeter, Microbial activity, Plant uptake

## Abstract

Sewage sludge is repeatedly applied as fertilizer on farmland due to its high nutrient content. This may lead to a significant increase of silver nanomaterials (AgNM) in soil over years. Therefore, our aim was to investigate the ecotoxicity and fate of AgNM under environmentally relevant conditions in outdoor lysimeters over 25 months. Two AgNM concentrations (1.7 and 8.0 mg/kg dry matter soil) were applied via sewage sludge into soil. In subsamples of the soil, incubated under laboratory conditions for 180 days, the comparability of outdoor and laboratory results regarding ecotoxicity was determined. The results from our long term lysimeter experiments show no detectable horizontal displacement in combination with very low remobilization to the percolate water. Thus, indicate that the sludge applied AgNM remains nearly immobile in the pathway between soils and leachate. However, Ag uptake to the roots of wheat and canola suggests that the chemical conditions in the rhizosphere induce AgNM remobilization from the incorporated sewage sludge even after two harvesting cycles. At the higher AgNM concentration a steady inhibition of the soil microflora was observed over 25 month in the lysimeter study, while there was no effect at the lower AgNM concentration. The results of the laboratory experiment reflect the findings of the lysimeter study and indicate that a risk assessment for AgNM based on data from laboratory tests is acceptable.

## Introduction

Silver nanomaterials (AgNM) have potent antimicrobial properties (Morones et al. 2005) making them suitable for diverse applications such as the manufacture of plastics, textiles, healthcare products, coatings and electrical appliances. The demand for such materials is rising, and thus increases the risk of AgNM entering the environment.

Studies have already confirmed the release of AgNM from textiles (Benn and Westerhoff 2008; Geranio et al. 2009), coatings (Kaegi et al. 2010) and consumer products (Cleveland et al. 2012). After their release from the product AgNM can be introduced into the environment via different routes. Released from e.g. textiles and cosmetics they may enter the environment mainly via the sewer system (Kaegi et al. 2013; Voelker et al. 2015). From the sewer system AgNM end up in sewage treatment plants where they can be retained in sewage sludge to around 90% (Kaegi et al. 2011; Schlich et al. 2013). The distribution of the nanomaterials into the terrestrial environment then commonly occurs via point-source accumulation in sewage sludge applied as agricultural fertiliser. Here, due to the AgNM antibacterial properties in the terrestrial environment soil microorganisms can be at great risk.

Ecotoxicological tests with AgNM under standardized laboratory conditions confirmed that pure AgNM may affect the biomass content by substrate-induced respiration, the enzyme activity of soils and ammonia oxidizing bacteria (Hänsch and Emmerling 2010; McGee et al. 2017; Schlich and Hund-Rinke 2015; Shin et al. 2012). There is also evidence that AgNMs affect the terrestrial environment, e.g. through the use of sewage sludge as agricultural fertilizer (Schlich et al. 2013). AgNMs that have been passed through a model sewage treatment plant, applied to soil via the sewage sludge and tested after 100–140 days, remained as toxic towards soil microorganisms as freshly-prepared AgNMs applied to soil and tested after 28 days.

Environmental fate studies have identified different environmental factors that influence the transport of AgNMs in soils (Aiken et al. 2011; Akaighe et al. 2011; Coutris et al. 2012; El Badawy et al. 2013; Sagee et al. 2012). Depending on the soil properties it was shown that there might be a high retention of the nanomaterials in soil (Cornelis et al. 2010; Coutris et al. 2012; Hoppe et al. 2014). Information about the fate of AgNM in soil has increased in the last years, and has been reviewed elsewhere (Cornelis et al. 2014). However, long-term studies under field conditions are still sparse. Cleveland et al. (2012) investigated AgNM in estuarine mesocosms and found that significant amounts of AgNM were taken up by organisms. Lowry et al. (2012) showed that PVP coated AgNM were retained in soil and sediment of a freshwater environment, however, small amounts of Ag remained bioaccessible. After 92 days of AgNM incubation in a cambisol (Refesol 01 A), the column remobilization potential was very low (Hoppe et al. 2015). Nonetheless, the remobilization of AgNM might occur under the varying physicochemical properties of the rhizosphere. In addition, Gardea-Torresdey et al. (2014) discussed major concerns about the possible trophic transfer of engineered nanoparticles (ENP) from soil to the human food chain.

All these studies presenting data about the ecotoxicity and fate of AgNM used mainly the pure nanomaterial and were performed under standardized laboratory conditions. As part of an OECD Expert Meeting on Ecotoxicology and Environmental Fate in Berlin (OECD Series on the Safety of Manufactured Nanomaterials No. 40 2014), the exposure pathway of nanomaterials to the natural environmental compartments was mentioned as an important process which may affect the environmental fate and effects of AgNMs. Application via sewage sludge was identified as an important entry pathway. It was mentioned that further research addressing these entry pathways and tests under varying conditions is needed. It was noted that the mobility of nanomaterials in soils is expected to be very limited (OECD Series on the Safety of Manufactured Nanomaterials No. 40 2014). For regulatory purposes, chemical assessment regarding the environment is usually based on laboratory tests performed under standardized test conditions but with limited environmental relevance. Such an approach is only justified if the results with the chemical substance can be linked to its behaviour in the environment. For nanomaterials information on the transferability of the results also with respect to a longer time span is still lacking.

Therefore, our aim was to investigate the ecotoxicity and fate of an AgNM applied via sewage sludge into soil under environmental relevant conditions in outdoor lysimeters. In addition, the ecotoxicity of the AgNM incubated under laboratory conditions was monitored to evaluate the comparability of outdoor and laboratory results. The fate of the AgNM in the outdoor lysimeter was investigated by regular measurement of the Ag content in leachate, in the top 40 cm of soil-sludge mixture, and in the lysimeters and in plant compartments.

## Materials and methods

### Test soil

The experiments were carried out using reference soil 01A (RefeSol; http://www.refesol.de/english/analysedaten.shtml), whose physicochemical properties are listed in Table 1. RefeSol 01A was chosen as a test soil to have a good comparability with a previous laboratory study with AgNM by Schlich et al. (2013). The soil is recognized by the German Federal Environment Agency for application in test procedures according to the German Federal Soil Protection Ordinance. It is recommended for studies in the scope of regulation. The phyisco-chemical properties of the soil met to the requirements of the OECD guidelines 216 and 217 (OECD Guideline 216 2000; OECD Guideline 217 2000) for the testing of chemicals, and therefore represent a soil important in the scope of regulation of substances. The soils were sampled in the field and directly filled into the lysimeter containers.

**Table 1:** Physicochemical properties of RefeSol 01A

Parameter	RefeSol 01A^1^
Soil group	Dystric Cambisol
Soil type	loamy sand
Sand [%]	73
Silt [%]	22
Clay [%]	5
pH (CaCl_2_) (before test initiation)	5.51 top layer (20 cm) 4.28 ground layer
Corg [%]	1.00
CEC_eff_ [mmolc/kg]	37.9
WHC_max_ [mL/kg]	292

(^1^arable land; *CEC* cation exchange capacity; *WHC*_*max*_ maximum water-holding capacity)

### Sewage sludge

Sewage sludge, fed with municipal sewage, was freshly gathered at the sewage treatment plant of Schmallenberg (Germany). Previous measurements showed that the silver concentration in the sewage sludge was on average 1.8 mg/kg dry matter (dm) sludge. The sewage sludge met the requirements of the German Sewage Sludge Ordinance (AbfKlärV §4 1992) regarding the metal content (lead, cadmium, chromium, copper, nickel, mercury, zinc) of sewage sludge used as fertilizer on agricultural land. For the application of AgNM the sewage sludge was sieved to particles smaller than 2 mm and then stored in a vessel under permanent moderate stirring and aeration (2.5 mg O_2_/L). AgNM was spiked into the sewage sludge by a ratio of AgNM to dry matter of sewage sludge sufficient to receive nominal concentrations of 2.5 mg/kg dm soil and 9.0 mg/kg dm soil after application via sewage sludge. The concentrations were chosen based on a previous study conducted under laboratory conditions (Schlich et al. 2013) to facilitate the comparability of both studies.

After addition of the AgNM into the sewage sludge, the sludge remained in the vessel for another 16 h under aeration and moderate stirring. This allowed transformation reactions of the AgNM with the surrounding media and adsorption with the sewage sludge.

According to the instructions of the local sewage treatment plant, water and sewage sludge were separated by decanting the water after addition of 3.5 mL/g dm sludge of a 0.2% cationic polyacrylamide solution (Sedifloc 154, Kemira Germany GmbH, Frankfurt) as flocculant.

### Silver nanomaterials

NM-300K was used as required by the OECD Sponsorship Programme (Organisation for Economic Co-operation and Development 2007). This is a colloidal silver dispersion with a nominal silver content of 10% (w/w) and a particle size of ~15 nm with a narrow size distribution (99%). A second particle size of 5 nm, which is much less abundant (1%), was identified by TEM (Klein et al. 2011). The particles are dispersed in a mixture of stabilizing agents (NM-300K DIS) comprising 4% (w/w) each of polyoxyethylene glycerol trioleate and polyoxyethylene sorbitan monolaurate (Tween-20) (Klein et al. 2011).

### Set up of lysimeters

**Fig. 1:**
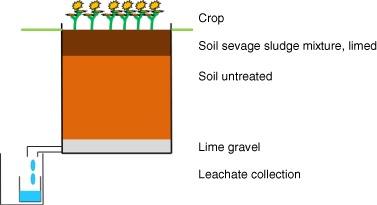
Lysimeter scheme and setup

The artificially filled lysimeters (0.9 × 0.9 × 0.9 m; ~1 t of dry matter soil) are cubic shaped and made of high grade stainless steel. The top 0.2 m soil of the lysimeters were treated with 200 g CaO to achieve a pH-value of an agricultural relevant level 6 months before the test initiation, followed by 0.5 m of untreated soil and 0.1 m lime gravel (*d* = 8 – 16 mm) (Fig. 1). The lysimeter experiment started at the end of May 2014. The sewage sludge was applied to the soil in two steps. First sewage sludge was mixed over 30 min with 25 kg dm soil (55% WHC_max_), taken from the top layer (20 cm) of the lysimeters, to receive a homogeneous mixture of soil and sewage sludge containing the AgNM. Afterwards the mixture of soil-sewage sludge was spread on the top of the soil in the lysimeters and mixed into the top 20 cm with a spade and a rake simulating the use of a plow.

**Fig. 2:**
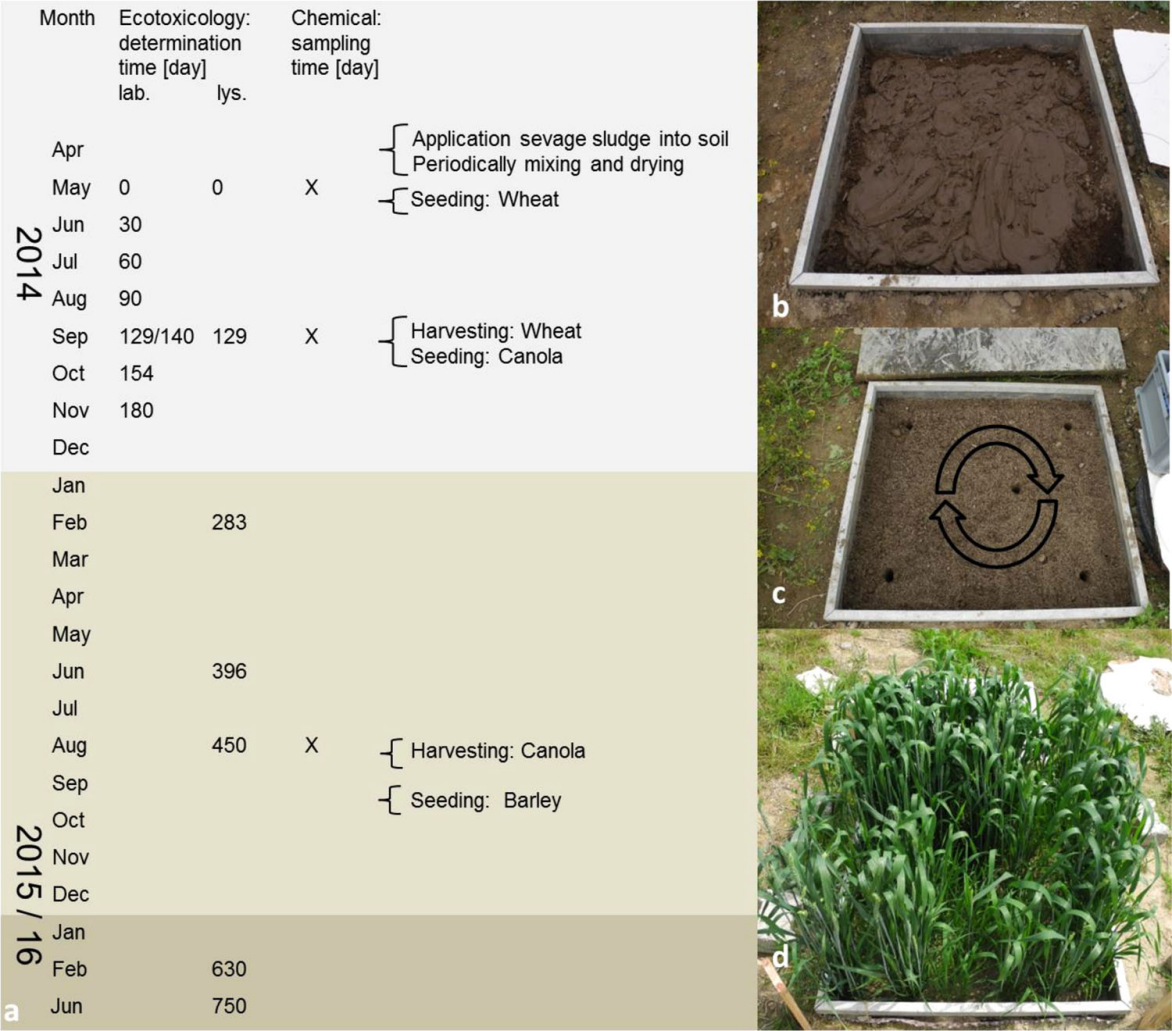
Time line of the lysimeter experiment (lys.) and laboratory experiment (lab.) with determination points of the ecotoxicological testing of the effect on ammonium oxidizing bacteria and the overall respiration activity **a** Photos of the lysimeter at different points: While sewage sludge application **b** sampling for chemical analysis, arrows are indicating twist between sampling events **c** and while growth period of wheat **d**

The lysimeters were cultivated with a series of agricultural crops relevant for sewage sludge fertilized fields (AbfKlärV §6 Absatz 1 1992), and adapted to the usual crop rotation in the surrounding region. The seeds were untreated for experimental purposes. In June 2014 summer wheat (*Triticum aestivum* ´Tybalt A‘ Saaten Union GmbH Isernhagen, Germany) was sown followed by winter canola (*Brassica napus* ‘Treffer‘ KWS Saat SE, Einbeck, Germany) in September 2014 and winter barley (*Hordeum vulgare* ‘ SY Typee‘ Syngenta, Maintal, Germany) in August 2015 (Fig. 2). The plant density was in accordance with the distributor´s recommendation. Plants were observed on a daily basis and irrigated with the same amount of tap water in dry periods. At harvest about 25% of the plants were taken including their roots, the remaining plants were cut 5 cm above the ground. The roots remained in the soil to prevent a removal of AgNM accumulated in the roots. The harvested plants were divided into root (if available), shoots and ear for wheat or root and shoot for canola.

After harvesting, five soil samples per lysimeter were taken using a soil sampler (Pürkhauer drilling stick) for the top 40 cm and divided in steps of 10 cm with a specific pattern (Fig. 2). The angle of the pattern was changed by 10 degrees between the different sampling events to prevent repeated drilling at the same spot. The drill-holes were filled with RefeSol 01A soil to prevent preferential flow. After sampling, the top 20 cm soil layer, including the roots, was mixed again and the subsequent crop was seeded.

The leachate was permanently collected and the volume was determined. If not directly analyzed, the leachate was stored at 4 °C until chemical analysis. Once per month fresh water samples were analyzed immediately after heavy rainfall to determine leaching of Ag through preferential flow. In addition water samples were taken regularly and preserved with 69% HNO_3_ suprapur (Carl Roth GmbH + Co. KG, Karlsruhe, Germany) before analysis.

The lysimeter study was conducted with one replicate as control containing unspiked sewage sludge and two treatments with AgNM applied to the lysimeter via sewage sludge.

One lysimeter per control and treatment was considered to be appropriate to achieve the goals of the present study. The volume of around 1 m^3^ of a sandy soil presents a homogeneous and integrating system. For the microbial determinations samples can be collected at different locations to consider potential inhomogeneity. Preferential flow is minimized by the large surface. Artificial conditions regarding growth of the plants are reduced by planting the same cultures on the area around the lysimeters. Since the aim of the study was to investigate the effects on soil microorganisms due to AgNM applied via sewage sludge into soil, no lysimeter containing only soil was included.

It had been shown that there was no effect on the soil microflora (ammonia oxidizing bacteria and microbial respiration) due to NM-300K DIS, the dispersant of NM-300K (Schlich et al. 2013). Therefore, no separate lysimeter containing only the dispersing agent, has been conducted.

Both the control and the AgNM treatments included sewage sludge from the same batch.

According to the German sewage sludge ordinance (AbfKlärV §4 1992), sludge can be applied on argicultural land at an amount of 5 tons/ha in 3 years. It was asumend that the complete amount of sewage sludge will be applied at once, which is the current practice. In addition, a soil depth of 20 cm (in accordance to the agricultural practice) and a soil bulk density of 1.5 g/cm^3^ (OECD Guideline 216 2000; OECD Guideline 217 2000) were assumed for the calculation of the amount of sewage sludge which could be applied to the soil.

### Climate and soil conditions

**Fig. 3:**
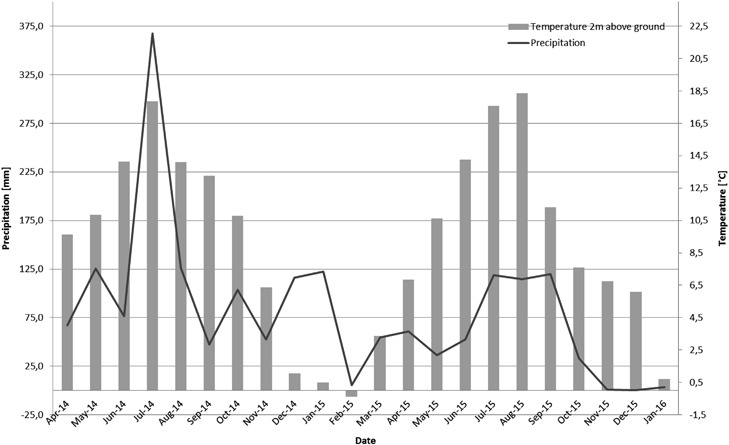
Precipitation and temperature (monthly mean) data during the outdoor lysimeter study

The average rainfall was about 96.2 mm per month ranging from 0.2 mm in December 2015 to 367.7 mm in July 2014. The temperature ranged from −0.4 °C in February 2014 and 18.4 °C in August 2015 (Fig. 3). The soil pH in the 20 cm top layer were between 6.0 and 5.3 for the control, 5.8 and 5.3 in the soil with the lower AgNM concentration and 5.7 and 5.4 in the soil with the high AgNM concentration after the application of the sewage sludge.

### Laboratory study

To compare outdoor data with laboratory data 6 kg dm soil of the soil-sludge-mixtures were taken out of the lysimeters directly after final mixing and before seeding. The mixtures were sieved to a particle size smaller than 2 mm and adjusted to 50% of the water holding capacity (WHC_max_). The incubation was performed in vessels with perforated lids at 20 ± 2 °C for 180 days. The soil-sludge-mixtures were turned every second week to avoid anaerobic conditions and the loss of water was adjusted with deionized water. After 0, 30, 60, 90, 129, 154 and 180 days effects on soil microorganisms were determined with two different ecotoxicological test systems.

### Ecotoxicological test systems

In accordance with the ECHA guidance on information requirements and chemical safety assessment (ECHA Guidance on information requirements and chemical safety assessment 2003) the C- and N-transformation are considered. Therefore, the investigations were performed following the OECD 217 (C-transformation) and ISO 15685 (N-transformation), which is more suitable for the testing of nanomaterials effects on ammonia oxidizing bacteria (Hund-Rinke and Schlich 2014).

In accordance with the ISO 15685 (ISO Guideline 15685 2012) effects on soil nitrifying bacteria the nitrite concentration was determined by a short-term potential ammonium oxidation test. The objective of this method is to measure the ammonia oxidation potential, which provides an indication of the size of the ammonia oxidizing bacterial population.

The carbon transformation (substrate induced respiration) was determined in accordance with the OECD 217 (OECD Guideline 217 2000). In this test glucose induced respiration rates (i.e. the mean of the quantities of carbon dioxide formed) were measured. The toxicity of AgNM to aerobic heterotrophic microorganisms utilizing available carbon sources is indicated by the glucose induced respiration rates, relative to the control.

### Chemical analysis of leachates, soil and plant material

The soil pH was measured in deionized water (water/soil = 5, v/v) after 2 h of extraction using a SenTix 41 electrode (WTW GmbH, Weilheim, Germany). According to Lowry et al. (2012), the dried and ground plant materials (roots, washed roots, shoots, pods, and grains) were digested with nitric acid (65% HNO_3_, Suprapur, Merck, Darmstadt, Germany). The amount of concentrated HNO_3_ was increased from 1.5 to 4 ml to enable a complete dissolution of the starch-containing grains. The Ag total concentration (Ag_total_) after HNO_3_ digestion was labeled as Ag_HNO3_. Aqua regia digestion (DIN 38414-7 1983) was applied to the ground soil samples. The Ag_total_ concentration after aqua regia digestion (ARD) was labeled as Ag_ARD_. According to DIN 38402-11 (2009), the leachates were filtered (0.45 µm, Graphic Controls, Buffallo, NY, USA) and acidified (HNO_3_ Suprapur; Carl Roth GmbH & Co. KG, Karlsruhe, Germany) immediately after sampling. The Ag_total_ concentration according to DIN 38402-11 (2009) was labeled as Ag_DIN38402_. Inductively coupled mass spectrometry (ICP-MS, 7500 Series, Agilent, Santa Clara, California, USA) and inductively coupled optical emission spectroscopy (ICP-OES, Ciros Vision, Spectro, Kleve, Germany) were used to determine the Ag_total_ contents in the leachates and in the digested soil and plant materials.

### Statistical analysis

Statistical analysis was carried out using ToxRatPro v2.10 software for ecotoxicity response analysis (ToxRat Solutions GmbH, Alsdorf, Germany) and SPSS 22.0.0.0 (IBM Corp., Armonk, USA). For the microbial tests Student´s t-test for homogeneous variances (one sided, (*p < 0.05; **p < 0.01; ***p < 0.001) was performed after prove of homogeneity of variances tested by Levene´s test (*α* = 0.05). In all figures, significant *p*-values are marked with asterisks.

The statistical analysis regarding the Ag_DIN38402_ concentrations in the lechates of the lysimeters were executed with IBM SPSS Statistics. The measurement data were not normal distributed which is why the non-parametric Mann-Whitney U test was applied. This test was used to assess whether the distributions of the Ag_DIN38402_ concentrations from the AgNM spiked lysimeters differed significantly from the control lysimeter.

## Results

### Fate

#### Soil Ag_ARD_ concentrations at the three samplings

**Fig. 4:**
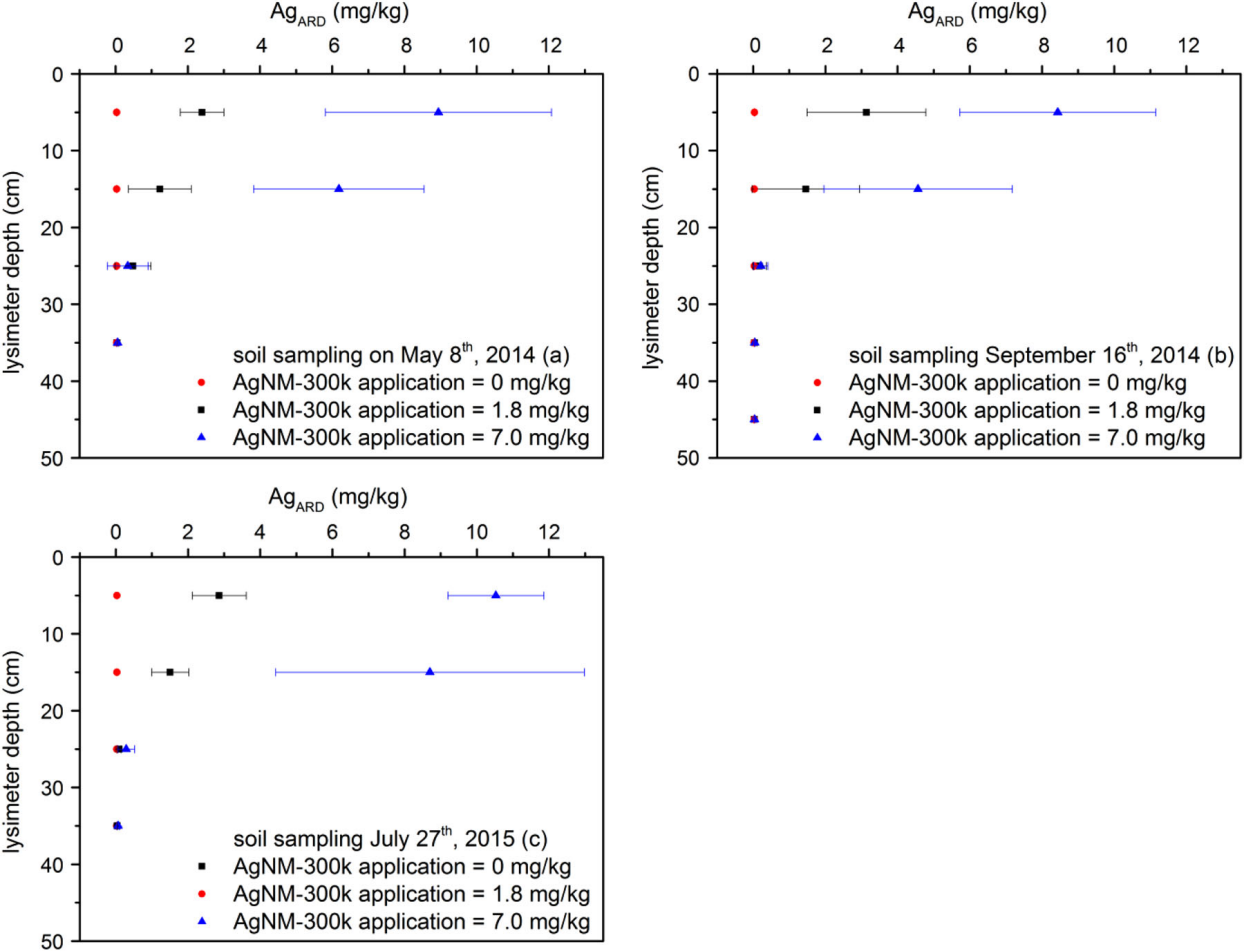
Ag total concentration after aqua regia digestion (Ag_ARD_) in the pooled samples of the uppermost four lysimeter horizons (0–10, 10–20, 20–30, 30–40 cm) after sludge application. The sludge was incorporated into the two uppermost horizons (0–20 cm). Error bars represent the standard deviation of five replicates

Figure 4 shows a timeline (May 8th, 2014; September 15th, 2014; July 28th, 2015) of the Ag_ARD_ concentrations in pooled samples (*n* = 5) of the four uppermost lysimeter horizons (0–10, 10–20, 20–30, and 30–40 cm). The Ag_ARD_ concentrations were low in all control samples (max. Ag_ARD_ = 0.04 ± 0.008 mg/kg dm soil). Thus, the measured Ag_ARD_ in the other lysimeters can be related to the applied AgNM. Compared to the control, no enhanced Ag_ARD_ concentration was detected in the pooled samples in the fourth horizon (max. Ag_ARD_ = 0.06 ± 0.03 mg/kg dm soil, 30–40 cm). However, the enhanced Ag_ARD_ concentrations in the third horizon (max. Ag_ARD_ = 0.5 ± 0.5 mg/kg dm soil, 20–30 cm, lysimeter 6) can probably be related to inaccuracy of the sampling by Pürckhauer or ploughing. This hypothesis is supported by the fact that the highest Ag_ARD_ concentration was found in the third horizon directly after sludge application (May 8th, 2014).

#### Ag_HNO3_ concentrations in plant material after harvesting

**Fig. 5:**
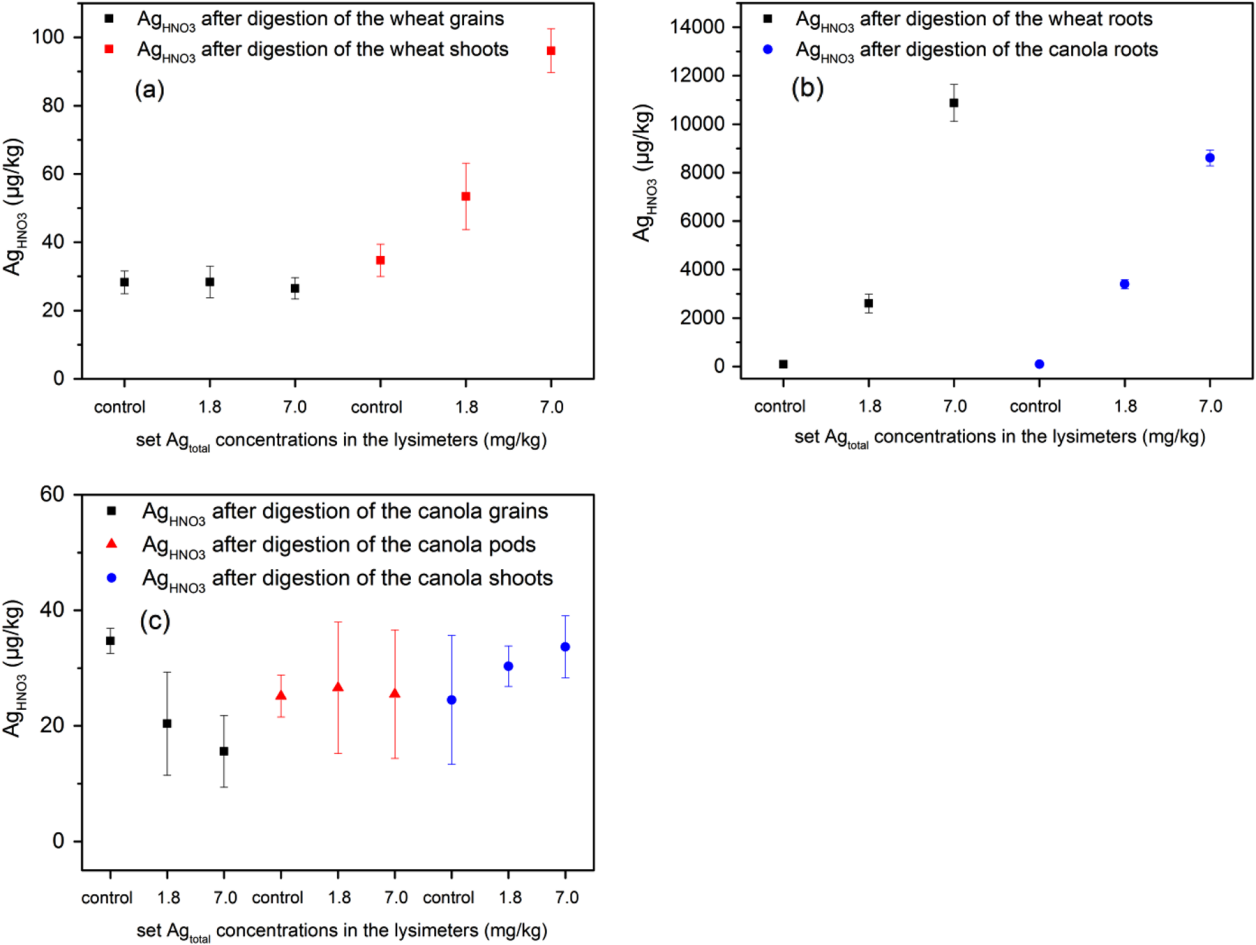
Ag total concentrations after HNO_3_ digestion in **a** the grains and shoots of the wheat (harvested September 16th, 2014); **b** the root material of the wheat (harvested September 16th, 2014) compared to the root material of the canola (harvested July 28th, 2015); **c** the grains, pods, and shoots of the canola (harvested July 27th, 2015). Error bars represent the standard deviation of three replicates

The Ag_HNO3_ concentrations in the wheat grains were on the same low level (<30 µg**/**kg) for all lysimeters (Fig. 5a), and in the range of the limit of quantification (LOQ). However, the Ag_HNO3_ concentrations in the shoots were enhanced in the lysimeters with 1.8 mg/kg dm soil and 7.0 mg/kg dm soil compared to the control. Figure 5c shows the Ag_HNO3_ concentrations in the grain, pod, and shoot of the canola samples. No accumulation of Ag_HNO3_ was detected in the above ground canola matrices. The washed root material of wheat and canola showed accumulation of Ag_HNO3_ in the roots from lysimeters with an AgNM concentration of 1.8 and 7.0 mg/kg dm soil compared to the control (Fig. 5b). The accumulation of Ag_HNO3_ in the roots of wheat and canola was on the same level for the lysimeter with the lower AgNM concentration of 1.8 mg/kg dm soil (mean Ag_HNO3_ = 2603 µg/kg (wheat) *vs*. mean Ag_HNO3_ = 3396 µg/kg (canola)) and the lysimeter with the higher AgNM concentration of 7.0 mg/kg dm soil (mean Ag_HNO3_ = 10879 µg/kg (wheat) *vs*. mean Ag_HNO3_ = 8611 µg**/**kg (canola)).

#### Ag_HNO3_ concentrations in leachates

From May 2014 to November 2015 the leachates were sampled from the three lysimeters (*n* *=* 96) after rainfall events. In general, the Ag_DIN38402_ concentrations do not provide information about the Ag species. The highest release was on a low level (max. Ag_DIN38402_ = 256 ng/L), and determined in a leachate of the lysimeter with an AgNM concentration of 7.0 mg/kg dm soil. The control showed a very low Ag release with an arithmetic mean of 24 ng/L (*n* *=* 32). According to the Mann-Whitney U test, the distribution of Ag_DIN38402_ concentrations released from the control and the lower AgNM concentration (arithmetic mean 25 ng/L, *n* *=* 33) show no significant deviations (*p* > 0.05). On the contrary, the distribution of Ag_DIN38402_ concentrations released from the control and the treatment with the higher AgNM concentration (arithmetic mean 56 ng/L, *n* *=* 31) of 7.0 mg/kg dm soil are significantly different according to the Mann-Whitney U test (*p* *<* 0.05). Thus, the application of 7.0 mg AgNM per kg soil generated a low continuous release of Ag to the leachates.

### Ecotoxicity

#### Potential ammonium oxidation

The activity of the ammonium oxidizing bacteria and the inhibition compared to the control for laboratory and lysimeter data is presented in Table 2.

**Table 2:** Actual data of the ammonium oxidizing bacteria activity [ng NO_2_-N /(g dm*h)^−1^] in the laboratory and lysimeter experiments and the subsequent inhibition [%] caused by AgNM applied via sewage sludge into the soil (*0.05 ≥ *p* ≥ 0.01; **0.01 ≥ *p* ≥ 0.001; ***p ≤ 0.001)

Date	Day	Control	1.8 mg/kg dm soil		7.0 mg/kg dm soil	
		Mean ± SD	Mean ± SD	Inhibitionto control	Mean ± SD	Inhibition to control
	[d]	[ng NO_2_-N /(g dm*h^−1^]	[ng NO_2_-N /(g dm*h^−1^]	[%]	[ng NO_2_-N /(g dm*h^−1^]	[%]
Laboratory experiment						
May-14	0	59.2 ± 8.54	53.6 ± 5.04	9.35	38.0 ± 8.38	35.9**
Jun-14	30	72.6 ± 5.58	82.2 ± 1.55	−13.3	78.1 ± 4.10	−7.59
Jul-14	60	78.5 ± 5.28	88.3 ± 7.58	−12.5	65.3 ± 6.38	16.8**
Aug-14	90	105.8 ± 7.46	119.0 ± 8.69	−12.5	76.5 ± 5.82	27.7***
Sep-14	129	61.8 ± 2.85	57.4 ± 15.0	7.00	25.3 ± 6.08	59.1***
Oct-14	154	38.2 ± 2.88	43.9 ± 5.68	−15.1	14.2 ± 1.83	62.9***
Nov-14	180	27.7 ± 6.55	21.5 ± 7.30	22.3	4.75 ± 0.58	82.9***
Lysimeter experiment						
May-14	0	59.2 ± 8.54	53.6 ± 5.04	9.35	38.0 ± 8.38	35.9**
Sep-14	129	120.6 ± 8.90	119.3 ± 11.5	6.38	71.4 ± 7.80	31.3***
Feb-15	238	35.3 ± 1.51	29.5 ± 1.98	16.3	28.3 ± 4.95	19.8*
Jun-15	396	84.8 ± 7.51	87.5 ± 8.73	−3.26*	68.5 ± 4.48	19.2*
Aug-15	450	76.7 ± 2.57	67.7 ± 2.67	11.8	62.2 ± 2.83	18.9**
Feb-16	630	66.7 ± 3.92	61.8 ± 5.99	7.45	43.2 ± 0.99	35.3***
June-16	750	66.6 ± 1.82	55.8 ± 2.32	16.2	33.1 ± 3.61	50.3***

In the laboratory experiment, the activity of the ammonium oxidizing bacteria was at 60 ng NO_2_-N/(g dm*h^−1^) in the control at test start. Afterwards, the activity in the control increased steadily until day 90 and then decreased until the end of the laboratory experiment. Due to the low activity in soil after 180 days it was decided to terminate the laboratory experiment.

The AgNM treatments were inhibited by 9% at an Ag concentration of 1.8 mg/kg dm soil and 36% in the higher concentration of 7.0 mg/kg dm soil at test start. The inhibition increased from 17% after 60 days up to 83% after 180 days. No significant inhibition compared to the control was found in the lower treatment (1.8 mg/kg dm soil) during the whole test period.

In the lysimeter experiment the ammonium oxidizing bacteria activity in the control showed a season dependent behaviour with higher activities in warmer periods and lower activities in colder periods of the year. Over the whole test duration the ammonium oxidizing bacteria were significantly inhibited by AgNM at the highest test concentration of 7.0 mg/kg dm soil. The inhibition in the treatment with 1.8 mg/kg dm soil was mainly below 10% inhibition and thus not significant. There were three sampling points (238, 450 and 750 days) with a slight but not significant difference to the control. The inhibition at an AgNM concentration of 7.0 mg/kg dm soil was 36% compared to the control at test start and decreased afterwards to around 20% at day 450. Afterwards, until day 630 the inhibition increased up to 35% and to 50% after 750 days.

#### Substrate induced respiration

Table 3 shows the laboratory and lysimeter activity data for the carbon transformation in terms of substrate induced respiration. In addition, the inhibition of the substrate induced respiration in the lysimeter and laboratory experiment is shown.

**Table 3:** Actual data of the respiration activity [mg O_2_/(100 g dm*h)^−1^] in the laboratory and lysimeter experiments and the subsequent inhibition [%] caused by AgNM applied via sewage sludge into the soil (*0.05 ≥ *p* ≥ 0.01; **0.01 ≥ *p* ≥ 0.001; ***p ≤ 0.001)

Date	Day	Control	1.8 mg/kg dm soil		7.0 mg/kg dm soil	
		Mean ± SD	Mean ± SD	Inhibition to control	Mean ± SD	Inhibition to control
		[mg O_2_/(100 g dm*h^−1^)]	[mg O_2_/(100 g dm*h^−1^)]	[%]	[mg O_2_/(100 g dm*h^−1^)]	[%]
Laboratory experiment						
May-14	0	0.84 ± 0.17	0.93 ± 0.42	−9.92	0.88 ± 0.30	−4.47
Jun-14	30	0.66 ± 0.01	0.64 ± 0.01	2.55	0.66 ± 0.07	−0.09
Jul-14	60	0.53 ± 0.01	0.54 ± 0.05	−1.47	0.48 ± 0.03	11.0*
Aug-14	90	0.40 ± 0.01	0.42 ± 0.01	−5.73	0.32 ± 0.02	20.7***
Sep-14	129	0.46 ± 0.02	0.43 ± 0.02	5.05	0.33 ± 0.03	26.7***
Oct-14	154	0.35 ± 0.02	0.33 ± 0.02	3.95	0.25 ± 0.00	27.9***
Nov-14	180	0.37 ± 0.03	0.36 ± 0.01	1.86	0.26 ± 0.01	30.1***
Lysimeter experiment						
May-14	0	0.84 ± 0.17	0.93 ± 0.42	−9.92	0.88 ± 0.30	−4.47
Sep-14	129	1.01 ± 0.13	0.94 ± 0.01	7.38	0.85 ± 0.02	12.6*
Feb-15	238	0.75 ± 0.03	0.70 ± 0.01	6.22	0.25 ± 0.01	66.3***
Jun-15	396	0.75 ± 0.00	0.88 ± 0.05	−16.9*	0.65 ± 0.02	13.0***
Aug-15	450	0.97 ± 0.03	0.81 ± 0.05	16.4	0.74 ± 0.11	24.1**
Feb-16	630	0.81 ± 0.07	0.74 ± 0.04	8.1	0.71 ± 0.02	11.5*
June-16	750	0.60 ± 0.02	0.62 ± 0.01	−2.30	0.44 ± 0.02	26.4**

The carbon transformation started with a value for the respiration activity of 0.84 mg O_2_/(100 g dm*h^−1^) in the control treatment. In the laboratory experiment the respiration activity of the control decreased by about the half until day 90 of the experiment and remained steady till day 154.

At test start and the following measurement both AgNM treatments had no adverse effects on the substrate induced respiration activity. After 60 days a significant inhibition of 11% was found at an AgNM concentration of 7.0 mg/kg dm which increased steadily up to an inhibition of 30% at test end. At an AgNM concentration of 1.8 mg/kg dm soil no significant difference of the respiration activity compared to the control was found over the whole test period.

In the lysimeter experiment the respiration activity in the control remained nearly stable over the whole course of the experiment. The lower AgNM concentration of 1.8 mg/kg dm soil resulted in an inhibition of less than 10% throughout the test expect after 450 days with a difference to the control of 16.4%, however, which was not statistically significant. At the end of the test after 750 days the inhibition again was below 10%. At a high AgNM concentration of 7.0 mg/kg dm soil the respiration activity was inhibited continuously from the second determination after 129 days. The inhibition increased from 13% (day 129) up to 24% after 450 days. After 630 days the inhibition decreased to 11.5% but was still statistically significant and at the last determination point of 750 days the inhibition again increased to 26%.

#### Tendency and comparability of results obtained in outdoor and laboratory experiments

Both experiments were in good agreement regarding inhibition determined for the ammonium oxidizing bacteria as well as inhibition for the substrate induced respiration. The inhibition observed in the laboratory experiments under standardized conditions was more pronounced than in the lysimeter experiment. In September 2014 for example when data were determined at the same determination point the inhibition of the high AgNM concentration of 7.0 mg/kg dm soil was two times higher in the laboratory experiment than in the lysimeter experiment for both ecotoxicological tests. Comparison of the activity in control treatments showed that the experiments in the laboratory were limited since the activity decreased over the whole test period of 180 days, whereas the activity in the lysimeter experiment varies and is in dependent on the climate and plant growth but is constant at a high level over the whole test period.

## Discussion

Our aim was to investigate the ecotoxicity and fate of an AgNM under environmental relevant conditions in outdoor lysimeters. In addition, the ecotoxicity of the AgNM incubated under laboratory conditions was observed to evaluate the comparability of outdoor and laboratory results of AgNM. To our knowledge, the data presented here gives the first insight into the long term behaviour and effect of AgNM in soil over a period of 25 months after application via sewage sludge.

Although the present study is complex regarding the test set up, there are still limitations. The treatment of the sewage sludge prior to application to soil and further treatments may lead to a change of speciation of AgNM and to an altered bioavailability, which has to be considered in the following discussion. Baalousha et al. (2015) have shown that e.g. a sulfidation of AgNM in medium appears very fast. Depending on the country and the subsequent usage, sewage sludge is digested under anaerobic conditions, but also stabilized under aerobic conditions or limed. In Germany sewage sludge is mainly stabilised in digestion tanks or under aerobic conditions (Wiechmann et al. 2012). In our experiment the AgNM were added into aerated sludge. A residence time of several hours allowed a transformation of AgNM under aerobic conditions.

### Fate

The ARD digestion of the lysimeter soil showed that the AgNM was not displaced in detectable amounts within the lysimeter profile over 15 months. Lowry et al. (2012) conducted long-term studies with a freshwater mesocosm and found that large amounts of the added AgNM remained in the soil and sediments. Durenkamp et al. (2016) found a low Ag release to the lechates of outdoor lysimeters treated with AgNM spiked sewage sludge. After 92 days of aging, more than 99 % of the applied AgNM was retained in the soil (Cambisol, Refesol 01 A) column whilst percolation with artificial rainwater (Hoppe et al. 2015). These results of laboratory column experiments and the presented results of the outdoor lysimeter experiments using the same AgNM (NM-300K) and soil (Refesol 01 A) showed a low AgNM mobility despite different experimental designs (application, duration). In addition, the low Ag_DIN38402_ concentrations (mean of 55 ng/L for the lysimeter with 7.0 mg/kg dm soil) in the leachate strengthen the implication of a low AgNM mobility in the lysimiter profil. Due to fast sulfidation processes the type of coating need not taken into account if the AgNM are applied to sewage sludge (Whitley et al. 2013). Hence, the presented findings might also be transferable to AgNM with other coatings than the sterically stabilized AgNM (NM-300K). The summarized results support the assumption that soils may be the final sink for engineered nanomaterials (ENM) (Cornelis et al. 2014; Pan and Xing 2012). However, other experimental approaches using AgNM to percolate soil columns showed a potential of AgNM migration which induce a potential risk of groundwater contamination (Liang et al. 2013). Thus, the test duration and the ENM application methods could determine the implication regarding the environmental risk assessment. In the case of AgNM, the application to sewage sludge and subsequent long-term incorporation into an outdoor lysimeter might represent the more realistic environmental pathway compared to the column percolation with the highly colloidal stable AgNM dispersion.

Nowack et al. (2015) recently discussed the lack of analytical tools to determine ENP in predicted environmental concentrations (PEC) which was also the case for the current study. Despite these critical arguments the results regarding the fate of AgNM are in line with the findings of other long-term studies (Durenkamp et al. 2016; Lowry et al. 2012). In general, no detectable horizontal displacement in combination with very low remobilization to the percolate water (mean Ag_total_ of 55 ng/L) indicate that the sludge applied AgNM remains nearly immobile in the pathway between soils and leachate. However, solely low concentrations of released nanomaterials, even a total concentration below 1 µg/L, could represent a high number of released particles.

Despite the low AgNM mobility at the interface between soil and leachate (Fig. 4), uptake of AgNM (measured as Ag_HNO3_) was shown for wheat and canola roots (Fig. 5b). This result implies that different physicochemical conditions in the rhizosphere could favor the remobilization and plant uptake of AgNM. The plant roots probably grow into the nutrient-rich sewage sludge, where the AgNM is incorporated. Hence, the root uptake of AgNM might be explained as a co-transport mechanism by nutrient uptake. The AgNM uptake by plants was reviewed by Gardea-Torresdey et al. (2014) who discussed the possibility of AgNM enrichment in the food chain. However, Stegemeier et al. (2015) conducted hydroponic exposure of alfalfa and indicated AgNM accumulation along the apoplasts of the roots, but only a low translocation to the shoot system. The current results likewise show root uptake but no accumulation of AgNM in the grains of wheat and canola (Fig. 5c), and thus, implying a root-shoot barrier for the AgNM. However, the application of sewage sludge to the horticultural production of root vegetables, which is restricted according to the German law (AbfKlärV §4 1992), could compromise the food chain.

In summary, the data show a low but continuous remobilization potential for the sludge-applied AgNM in the investigated cambisol. On the one hand, enhanced release to the leachate was only found for the highest AgNM concentration lysimeter. This approach represents a worst case scenario with AgNM concentrations above the PEC calculated by Nowack et al. (2015). On the other hand, root uptake indicates that the chemical conditions in the rhizosphere induce AgNM remobilization from the incorporated sewage sludge even after two harvesting cycles. Thus, continuous negative effects on soil organisms in the AgNM loaded rhizosphere cannot be excluded.

### Ecotoxicity

#### Effect of AgNM in an outdoor lysimeter experiment

The measured concentrations of the outdoor lysimeters of 7.0 mg/kg dm soil caused a continuous inhibition of the ammonium oxidizing bacteria and microbial biomass over the entire test period of 22 months. The AgNM was more toxic towards ammonium oxidizing bacteria than to aerobic heterotrophic microorganisms (measured via substrate-induced respiration). This was demonstrated by a lower sensitivity of the study endpoint respiration activity in agreement with previous laboratory studies reporting the higher sensitivity of ammonium oxidizing bacteria (Hänsch and Emmerling 2010; Hund-Rinke and Schlich 2014; Schlich et al. 2013).

In the outdoor lysimeter experiment at an AgNM concentration of 1.8 mg/kg dm soil a statistically significant difference of the activity of the ammonium oxidizing bacteria and the substrate induced respiration rates compared to the control was not found throughout the whole test period. In two outdoor experiments, both stronger and weaker effects compared with our experiment were found (Colman et al. 2013; Durenkamp et al. 2016).

In an experiment by Colman et al. (2013) significant effects of AgNM on plants and microorganisms at a lower concentration of 0.14 mg/kg dm soil were determined in an outdoor mesocosm experiment. The AgNM had a primary particles size of 21 nm (TEM analysis) and a polyvinylpyrrolodine (PVP)-coating. The application was performed via sewage sludge applied to the top of soil (surface mineral soils from the floodplain: 63.5% sand, 10.5% silt, 26% clay) already having plant growth. An impact of the AgNM on the activity of the soil microbial extracellular enzymes leucine amino peptidase and phosphatase and the microbial biomass was found (Colman et al. 2013). The different effect sizes can be explained by differences in the experimental approach as in the treatment of the sewage sludge, the application of sewage sludge to soil or in different soil properties. In contrast to Colman et al. (2013), in our experiment the sewage sludge first was dewatered using a flocculant (cationic polyacrylamide solution), which had no effect on the soil microflora as it was already shown in earlier studies. This process is an often performed step in a sewage treatment plant to reduce the volume and weight for the transportation of the sewage sludge to agricultural areas and thus enhanced the environmental relevance of the experiment (Personal communication; operator of the sewage treatment plant of Schmallenberg, Germany). In addition, different amounts of sewage sludge were incorporated into the soil. Colman et al. (2013) used 200 g of Class A biosolids mixed in deionized water for 80 kg of mineral soil in the outdoor mesocosm. In contrast, the first step in the present experiment 405 g sewage sludge after water removal containing AgNM were mixed into 25 kg dm soil of the lysimeter and this mixture was then mixed into the topmost 20 cm of the lysimeters. This led to a sludge-soil-ratio of 5t/ha which can be applied to agricultural used land according to the German Sewage Sludge Ordinance (AbfKlärV §6 Absatz 1 1992) within 3 years. Furthermore, the organic matter content or the texture of the test soil can have a strong influence on the toxic effect of AgNM (Collin et al. 2014; Cupi et al. 2015; Schlich and Hund-Rinke 2015). In the present experiment a sandy loam was used as test soil sieved ≤2 mm before the soil was filled into the lysimeters, whereas Colman et al. (2013) used a mineral soil from a floodplain with a high clay content (26%) sieved to 10 mm before test start.

The effect and fate of AgNM on the soil microbial community structure and function after the application of AgNM via sewage sludge in outdoor lysimeter (0.11 × 0.23 m) was also investigated by Durenkamp et al. (2016) over 6 months. They used an AgNM concentration of 140 mg/kg dm soil for their experiment which was applied via sewage sludge into a sandy soil. Soil and sewage sludge were mixed at a ratio of 58:42%. The AgNM lead to changes in the nitrogen cycling and to a decrease in CH_4_ emission. It was also shown, that fungal communities were affected by the AgNM after application via sewage sludge into soil, which may influence the decomposition of organic matter and have an impact on the nutrient cycling and subsequently on plant growth (Durenkamp et al. 2016). The results confirm that soil microorganisms involved in the nitrogen cycle are the most sensitive to AgNM.

#### Laboratory versus outdoor experiment

Another issue of the present experiment was to evaluate the comparability of the ecotoxicity of the AgNM in laboratory experiments and in an outdoor lysimeter experiment. The risk assessment e.g. in accordance to REACH (Registration, Evaluation, Authorisation and Restriction of Chemicals) regarding the ecotoxicology of the test item is based on effect concentrations, which were determined in laboratory experiments (European Chemicals Agency 2008). If, based on this evaluation, a risk to the environment cannot be excluded the data available must be refined through increasingly more complex experiments. The utilisation of outdoor lysimeter experiments represents such a test system. For a sufficient risk assessment the results obtained in the laboratory must present a comparable or higher sensitivity towards the test item than in the outdoor experiment to exclude a risk to the environment. In our experiment it has been shown that the effects on soil microorganisms in laboratory tests (I) were slightly higher than in the outdoor experiment and (II) that the intensity of the toxicity steadily increased in the laboratory experiment, while it remained almost constant in the outdoor experiment.

The reasons for the different results regarding an increasing or constant inhibition in the outdoor and laboratory experiment remain unclear. An explanation could be the leaching of AgNM from the soil. However, due to the high retention of AgNM in soil, as shown in the fate part of the experiment, this can be eliminated (Cornelis et al. 2014; Hoppe et al. 2015). Moreover, translocation of AgNM in the plant roots (Lowry et al. 2012; Stegemeier et al. 2015) even over a test period of over 2 years and reduction of the AgNM concentration due to the uptake into the plant biomass cannot explain the effects.

The actual data of all tests indicated a slightly higher respiration activity (substrate-induced respiration) and activity of the ammonium oxidizing bacteria in the field experiment than in the laboratory experiments, due to the plant growth and the concomitant presence of root exudates, which can increase the microbial activity in the rhizosphere in the field experiments. Therefore, the duration of laboratory experiments addressing microbial activity is limited (OECD Guideline 217 2000). The loss of nutrients from test start until test end due to the incubation under laboratory conditions and the subsequent decrease of the substrate-induced respiration rates and the activity of the ammonium oxidizing bacteria lead to a limited test duration of 180 days for the laboratory experiments.

The results of the laboratory experiment for ammonium oxidizing bacteria are comparable with those from Schlich et al. (2013) on the effects of AgNM on soil microorganisms after application via sewage sludge, which were the basis for the tests presented here. By this the reproducibility of effects caused by AgNM is demonstrated and is a prerequisite for the comparison of laboratory and outdoor experiments. The effect of AgNM applied via sewage sludge increased steadily over the test duration and after 140 days was in a comparable range with the results obtained with pristine AgNM. The findings suggest that the transformation of AgNM due to a reaction with the surrounding media does not completely detoxify the AgNM, which was bioavailable.

Furthermore, in several studies, the effect of pristine AgNM on soil microorganisms without applying AgNM via sewage sludge in soil was investigated (Hänsch and Emmerling 2010; Schlich and Hund-Rinke 2015; Shin et al. 2012). Effects induced by the pristine AgNM were in a comparable concentration range as in the present study in which the AgNM were applied via sewage sludge to the soil.

The findings clearly demonstrate that a risk assessment for AgNM as described in Voelker et al. (2015) based mainly on laboratory data is a suitable way at least for the terrestrial environment. The effect on the ammonium oxidizing bacteria in the outdoor lysimeter experiment is lower, nevertheless, even after 25 months an effect can be detected which is comparable with results obtained under laboratory conditions.

## Conclusions

In practice, sewage sludge is repeatedly applied as fertilizer on farmland due to its high content of nutrients and therefore may lead to a significant increase of AgNM in soil over years inducing a steady adverse effect on the terrestrial ecosystem. The results from our long term lysimeter experiments indicate that AgNM, which enter the soil via sewage sludge, will partly be remobilized in the rhizosphere and translocated to the roots of wheat and canola. Hence, the AgNM might be bioavailable over several years.

The results also indicate that a risk assessment for AgNM based on data from laboratory tests can be acceptable due to the high comparability with the obtained results of the outdoor experiments. Nevertheless, our results showed that ecotoxicological data from laboratory tests performed in accordance to actual guidelines and used for risk assessment represent a worst case scenario. These results of laboratory tests are applicable to predict the impact of AgNM in the natural environment as proven by the results of our outdoor lysimeter experiment.
